# *XIST*-Promoter Demethylation as Tissue Biomarker for Testicular Germ Cell Tumors and Spermatogenesis Quality

**DOI:** 10.3390/cancers11091385

**Published:** 2019-09-17

**Authors:** João Lobo, Sandra P. Nunes, Ad J. M. Gillis, Daniela Barros-Silva, Vera Miranda-Gonçalves, Annette van den Berg, Mariana Cantante, Rita Guimarães, Rui Henrique, Carmen Jerónimo, Leendert H. J. Looijenga

**Affiliations:** 1 Princess Máxima Center for Pediatric Oncology, Heidelberglaan 25, 3584 CS Utrecht, The Netherlandsa.j.m.gillis@prinsesmaximacentrum.nl (A.J.M.G.);; 2 Department of Pathology, Portuguese Oncology Institute of Porto (IPOP), R. Dr. António Bernardino de Almeida, 4200-072 Porto, Portugal; marianacantantecf@gmail.com (M.C.); ritaguimaraes.apct@gmail.com (R.G.); henrique@ipoporto.min-saude.pt (R.H.); 3 Cancer Biology and Epigenetics Group, Research Center of Portuguese Oncology Institute of Porto (GEBC CI-IPOP) and Porto Comprehensive Cancer Center (P.CCC), R. Dr. António Bernardino de Almeida, 4200-072 Porto, Portugal; sandra22nunes@gmail.com (S.P.N.); daniela.barros.silva94@gmail.com (D.B.-S.); verammgoncalves@hotmail.com (V.M.-G.); carmenjeronimo@ipoporto.min-saude.pt (C.J.); 4 Department of Pathology and Molecular Immunology, Institute of Biomedical Sciences Abel Salazar, University of Porto (ICBAS-UP), Rua Jorge Viterbo Ferreira 228, 4050-513 Porto, Portugal; 5 Department of Pathology, Lab. for Exp. Patho-Oncology (LEPO), Erasmus MC-University Medical Center Rotterdam, Cancer Institute, Be-432A, PO Box 2040, 3000 CA Rotterdam, The Netherlands

**Keywords:** molecular biomarkers, testicular germ cell tumors, spermatogenesis, methylation, *XIST* promoter

## Abstract

Background: The event of X chromosome inactivation induced by *XIST*, which is physiologically observed in females, is retained in testicular germ cell tumors (TGCTs), as a result of a supernumerary X chromosome constitution. X chromosome inactivation also occurs in male germline, specifically during spermatogenesis. We aimed to analyze the promoter methylation status of *XIST* in a series of TGCT tissues, representative cell lines, and testicular parenchyma. Methods: Two independent cohorts were included, comprising a total of 413 TGCT samples, four (T)GCT cell lines, and 86 testicular parenchyma samples. The relative amount of methylated and demethylated *XIST* promoter fragments was assessed by quantitative methylation-specific PCR (qMSP) and more sensitive high-resolution melting (HRM) methylation analyses. Results: Seminomas showed a lower amount of methylated *XIST* fragments as compared to non-seminomas or normal testis (*p* < 0.0001), allowing for a good discrimination among these groups (area under the curve 0.83 and 0.81, respectively). Seminomas showed a significantly higher content of demethylated *XIST* as compared to non-seminomas. The percentage of demethylated *XIST* fragment in cell lines reflected their chromosomal constitution (number of extra X chromosomes). A novel and strong positive correlation between the Johnsen’s score and *XIST* demethylation was identified (r = 0.75, *p* < 0.0001). Conclusions: The X chromosome inactivation event and demethylated *XIST* promoter are promising biomarkers for TGCTs and for assessing spermatogenesis quality.

## 1. Introduction

Germ cell tumors (GCTs) are remarkable developmental cancers, meaning that they recapitulate the various steps of embryonic and germ cell development, reflecting the main characteristics of their cells of origin [1]. They can arise not only in the gonads (testis and ovary) but also in extragonadal sites along the midline of the body, and they can afflict a wide age range of patients, from children to young adults. Epigenetic deregulation plays an important role in their genesis [2], which is in line with this developmental model, since few recurrent mutations are found in these neoplasms. Instead, a “genvironmental” model underlies tumorigenesis [3], and helps to explain the epidemiology and classification of these tumors (from type 0 to type VI tumors) [1,4,5].

Among the heterogeneous group of GCTs, the testicular germ cell tumors (TGCTs) are the most common. The type II tumors are the most frequent and clinically relevant [6]. They derive from a common precursor lesion, germ cell neoplasia *in situ* (GCNIS), and are divided into the following two major and relevant categories, with distinct clinical behavior: the seminomas (SEs) and the various non-seminoma (NS) subtypes, including embryonal carcinoma (EC), (postpubertal-type) yolk sac tumor (YST), choriocarcinoma (CH), and (postpubertal-type) teratoma (TE) [7]. Specifically, for these tumors, developmental biology has an invaluable impact by driving the identification of relevant disease biomarkers. On the one hand, the currently available serum markers of the disease, (alpha fetoprotein [AFP] and human chorionic gonadotropin subunit beta [β-HCG]), normally expressed during embryogenesis, show important limitations. On the other hand, the pluripotency factors constitute the basis of disease classification but are only informative based on tissue sample investigations. Finally, and more recently, a set of embryonic microRNAs that regulate development have shown outstanding performance as liquid biomarkers, bringing them closer to clinical introduction [8].

In this developmental perspective, another specific event of these neoplasms that might be used as a molecular biomarker is the X chromosome inactivation (or lyonization, described by Mary Lyon [9]). Indeed, this biological process occurs physiologically in females (XX) and is limited to germ cells in the male, is in fact recapitulated in all variants of type II TGCTs, as they show a gain of the X chromosome (due to whole genome duplication as an early pathogenetic event). This process of X chromosome inactivation involves *XIST*, a long non-coding RNA (lncRNA) which facilitates this process on extra X chromosomes by inducing a complex cascade of chromatin changes, including methylation and histone modifications [10,11]. *XIST* expression was indeed documented in these tumors (particularly in SEs), and allowed for the tolerance of supernumerary X chromosomes [12]. Later, Kawakami et al. [13] studied in detail the *XIST* promoter (by bisulfite sequencing, followed by conventional polymerase chain reaction (PCR) with primers specific for the methylated and demethylated *XIST* promoter), finding that region IV was frequently demethylated in TGCTs (again, especially in SEs) [14]. In addition, importantly, they demonstrated no evidence of this demethylated fragment in somatic male cancers. Exploring these findings in larger cohorts, with quantitative and more sensitive methodology and in liquid biopsy setting could be of clinical value, complementing other biomarkers for diagnosis and follow-up of type II TGCT patients [15]. To date, no such studies have been pursued.

Infertility is a frequent side effect from cancer treatments that severely impacts the quality of life of cancer survivors. Type II TGCTs, and GCTs in general, have an overall good prognosis and afflict mainly young adults, with long life expectancy, so fertility issues are a major concern [16]. Studies demonstrated that *XIST* is re-expressed transiently, in waves, during spermatogenesis around the time cells enter meiosis [17,18]. It is believed that X chromosome inactivation constitutes a protecting mechanism necessary for normal spermatogenesis, forming the so-called “XY body” and avoiding aberrant crosslinks among the sex chromosomes, with cells failing to do so being eliminated by apoptosis [19]. One of the ways of evaluating the efficiency of spermatogenesis is by applying the Johnsen’s score on histological samples of testicular parenchyma [20]. However, this analysis is laborious and, importantly, not very reproducible and subjected to sampling issues [21]. Novel markers for reliably estimating the quality of spermatogenesis in male individuals allowing for better selection for testicular sperm extraction (TESE) or other interventions are desirable, although not present so far [22].

The aim of this work is to explore the role of demethylated and methylated *XIST* promoter as a candidate biomarker for both TGCTs as well as extensiveness of spermatogenesis.

## 2. Results

### 2.1. Methylated XIST Promoter in TGCTs

**Figure 1:**
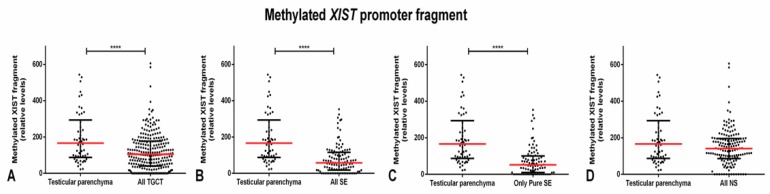
Methylated *XIST* fragment relative amounts in testicular germ cell tumors and testicular parenchyma of the discovery cohort. Relative amounts of the methylated *XIST* fragment among testicular parenchyma samples and (**A**) all testicular germ cell tumors (TGCTs), (**B**) all seminomas (SEs), (**C**) only pure SEs, and (**D**) all non-seminomas (NSs). **** indicate *p* < 0.0001. Abbreviations: NS—non-seminoma; SE—seminoma; TGCT—testicular germ cell tumor.

First, we first focused on the methylated *XIST* promoter fragment, specifically in our discovery cohort (clinicopathological features depicted in Supplementary Table S1). Considering all 250 TGCT tumor samples, they showed a significantly lower relative amount of methylated *XIST* fragment as compared to testicular parenchyma samples (*p* < 0.0001). This difference occurred at the expense of SEs, being more remarkable when selecting all SE components (pure and present in mixed tumors) (*p* < 0.0001) and even more when selecting only the pure SE samples (*p* < 0.0001). In fact, no significant differences in the relative amount of methylated *XIST* were found as compared with NS samples and the testicular parenchyma cohort (Figure 1).

**Figure 2:**
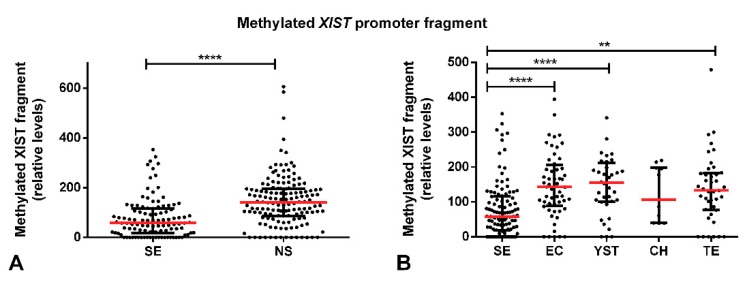
Methylated *XIST* fragment relative amounts among various tumor subtypes of the discovery cohort. Relative amounts of the methylated *XIST* fragment in (**A**) seminoma and non-seminoma and (**B**) all histological subtypes. **** indicates *p* < 0.0001 and ** indicates *p* < 0.01. Abbreviations: CH—choriocarcinoma; EC—embryonal carcinoma; NS—non-seminoma; SE—seminoma; TE—postpubertal-type teratoma; YST—postpubertal-type yolk sac tumor.

Moreover, SEs exhibited a significantly lower amount of methylated *XIST* fragment as compared with NS components (*p* < 0.0001) and there were also significant differences in the amount of methylated *XIST* fragment among the various individual tumor subtypes (*p* < 0.0001). When each of the five histological tumor components were considered individually, SE subtype showed a lower amount of this fragment in comparison with EC and YST (adjusted *p*-value < 0.0001) and TE (adjusted *p*-value < 0.01) (Figure 2).

**Figure 3:**
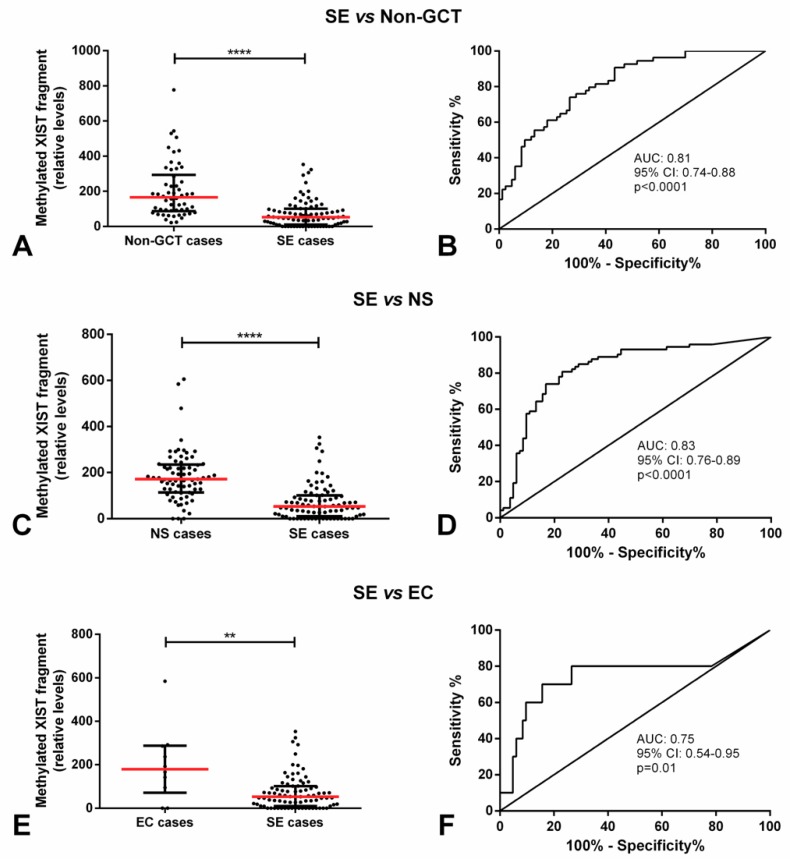
Methylated *XIST* fragment relative amounts in seminoma patients of the discovery cohort. Relative amounts of the methylated *XIST* fragment in pure seminoma patients and (**A**) no evidence of TGCT, respective receiving operator characteristic (ROC) curve in (**B**); (**C**) NS, respective ROC curve in (**D**); and (**E**) only pure embryonal carcinoma (EC), respective ROC curve in (**F**). **** indicates *p* < 0.0001 and ** indicates *p* < 0.01. Abbreviations: AUC—area under the curve; EC—embryonal carcinoma; GCT—germ cell tumor; NS—non-seminoma; SE—seminoma.

The relative amount of the methylated *XIST* fragment in SE cases was significantly lower as compared to patients with no GCT pathology, cases with NS, and specifically cases with pure EC (*p* < 0.0001, *p* < 0.0001, *p* < 0.01, respectively). Methylated *XIST* fragment relative amount allowed for a good discrimination among these groups, with an area under the curve (AUCs) of 0.81, 0.83 and 0.75, respectively (Figure 3). This allowed for a good performance in the discrimination, with sensitivity, specificity, and accuracy consistently above 70% (Table 1).

No significant correlation was found between the relative amount of methylated *XIST* fragment and the age of non-GCT, SE, and NS patients, respectively (*p* = 0.855, *p* = 0.064, and *p* = 0.761). Moreover, we found no significant association with patients’ stage, metastatic dissemination or the International Germ Cell Cancer Collaborative Group (IGCCCG) category (data not shown), which is in line with publicly available TCGA data, showing hypermethylation of the *XIST* promoter both in SE as well as in NS. These results are likely biased due to non-tumor contamination as reported before, found to be present both in SE as well as in NS [23]. No results on demethylation specifically have been reported in the TCGA dataset [24].

### 2.2. Demethylated XIST Promoter in TGCTs, GCNIS, and in (T)GCT Cell Lines

We then focused on the demethylated *XIST* promoter fragment, which we studied in two independent cohorts. Considering all tumor samples in the discovery cohort, no significant differences in the relative amount of the demethylated fragment were depicted between tumor samples (as a whole or grouped as SE/NS) and the testicular parenchyma samples (Supplementary Figure S1).

**Figure 4:**
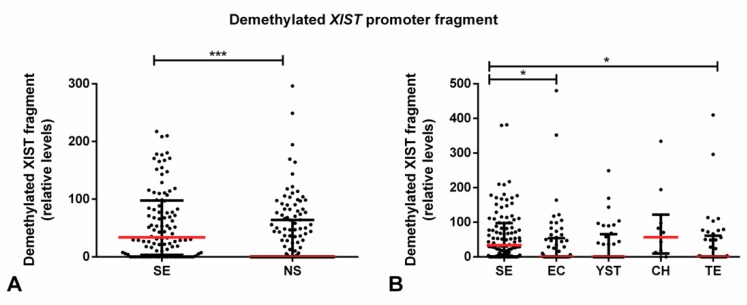
Demethylated *XIST* fragment relative amounts among various tumor subtypes of the discovery cohort. Relative amounts of the demethylated *XIST* fragment in (**A**) seminoma and non-seminoma and (**B**) all histological subtypes. *** indicates *p* < 0.001, * indicates *p* < 0.05. Abbreviations: CH—choriocarcinoma; EC—embryonal carcinoma; NS—non-seminoma; SE—seminoma; TE—postpubertal-type teratoma; YST—postpubertal-type yolk sac tumor.

However, SE samples were found to exhibit a higher relative amount of the demethylated *XIST* fragment as compared with NS (*p* < 0.001), with the same occurring when compared individually to EC and TE tumor subtypes (adjusted *p*-value < 0.05, Figure 4).

Again, when focusing on the TGCT cases, SE showed a significantly higher amount of *XIST* demethylated fragment as compared to EC (*p* < 0.01, Figure S2). Similar to the methylated fragment, we found no significant association between the amount of demethylated XIST and patients’ stage, metastatic dissemination or the IGCCCG category (data not shown).

**Figure 5:**
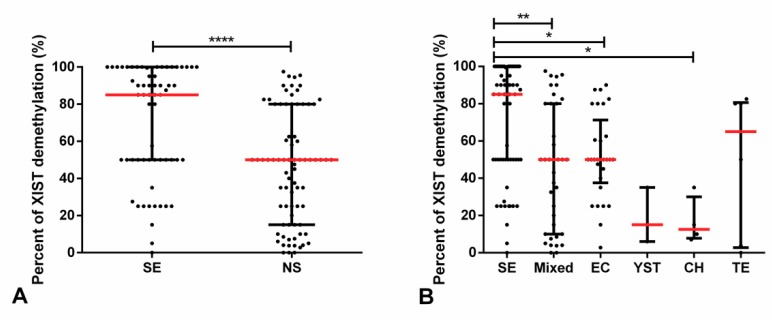
Demethylated *XIST* fragment percentage among various tumor subtypes (validation cohort). (**A**) Percentage of demethylated *XIST* fragment in seminoma and non-seminoma and (**B**) percentage of demethylated *XIST* fragment among all histological subtypes. **** indicates *p* < 0.0001, *** indicates *p* < 0.001, ** indicates *p* < 0.01, and * indicates *p* < 0.05. Abbreviations: CH—choriocarcinoma; EC—embryonal carcinoma; NS—non-seminoma; SE—seminoma; TE—postpubertal-type teratoma; YST—postpubertal-type yolk sac tumor.

We then pursued validation and further extension of our findings in a second (validation) cohort (clinicopathological details of this second cohort available in Supplementary Table S2). The percentage of demethylated *XIST* was significantly higher in SE as compared with NS (*p* < 0.0001). Additionally, there were also significant differences in the percentage of *XIST* demethylation among individual subtypes (*p* < 0.0001). SE displayed significantly higher percentage of demethylation of *XIST* promoter as compared with mixed tumors (adjusted *p*-value 0.0015), EC (adjusted *p*-value 0.0439), and CH (adjusted *p*-value 0.0212) (Figure 5). Importantly, some degree of demethylation of *XIST* promoter was able to be detected in 137 of 146 (93.8%) TGCTs; only nine tumor samples (three SE and six NS, including three mixed tumors, one pure EC, one pure YST, and one pure TE) showed absence of demethylated *XIST*.

**Figure 6:**
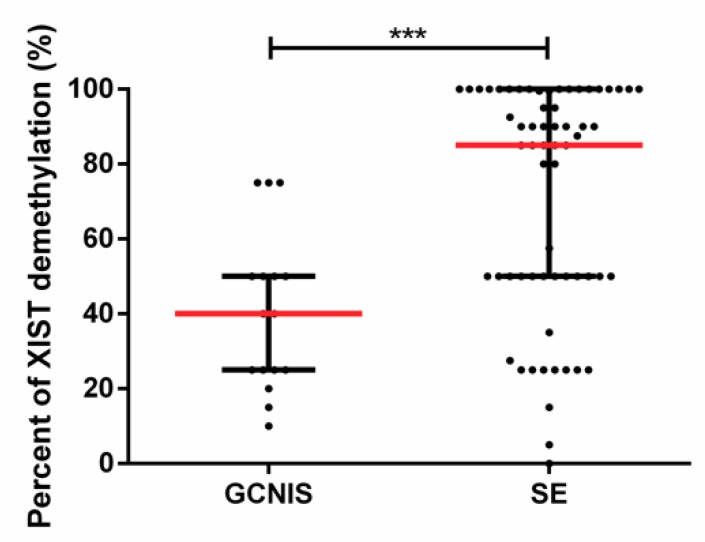
Demethylated *XIST* fragment percentage in germ cell neoplasia *in situ* (GCNIS) samples. Percentage of demethylated *XIST* fragment in GCNIS and seminoma. *** indicates *p* < 0.001 and ** indicates *p* < 0.01. Abbreviations: GCNIS—germ cell neoplasia in situ; SE—seminoma.

Moreover, demethylated *XIST* was also detected in 16 of 17 (94.1%) GCNIS lesions. Despite the reported crescent *XIST* expression in cases with increasingly more GCNIS content [12], we did not find the same positive correlation between the extent of GCNIS and the degree of *XIST* demethylation (data not shown). However, the percentage of demethylated *XIST* was significantly lower in GCNIS lesions as compared with overt SE (*p* = 0.0001, Figure 6).

**Figure 7:**
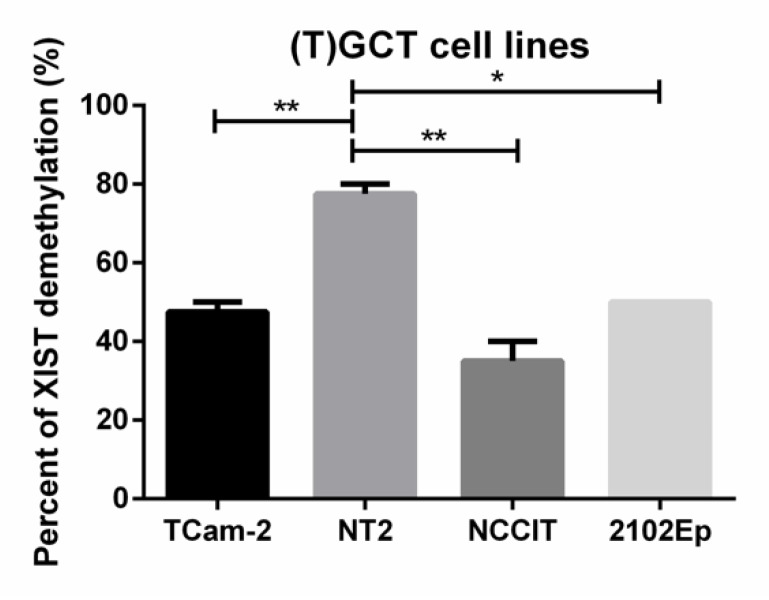
Demethylated *XIST* fragment percentage in germ cell tumor cell lines. ** indicates *p* < 0.01 and * indicates *p* < 0.05. Abbreviations: TGCT—testicular germ cell tumor.

Regarding (T)GCT cell lines, demethylated *XIST* was detected in all four cell lines. The percentage of demethylated *XIST* was significantly higher in NT2 as compared with the remaining cell lines (adjusted *p*-values of 0.0079, 0.0021, and 0.0108 as compared with TCam-2, NCCIT, and 2102Ep, respectively) (Figure 7). This is in line with previous karyotype findings reported before with NT2 cells showing three X chromosomes compared to two in 2102Ep and NCCIT.

### 2.3. Demethylated XIST Promoter in Testicular Parenchyma

**Figure 8:**
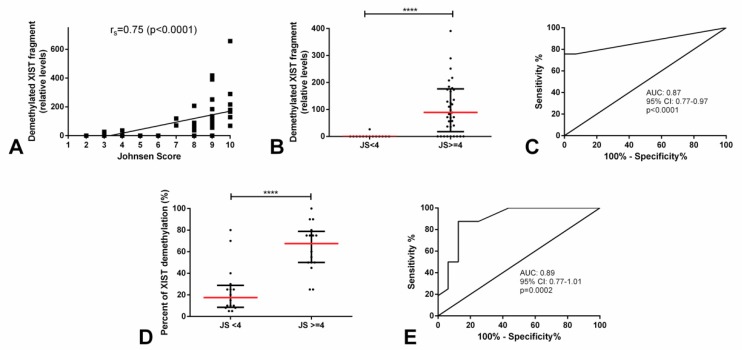
Demethylated *XIST* fragment relative amounts in testicular parenchyma with various Johnsen’s scores (discovery and validation cohorts). (**A**) Correlation between Johnsen’s score and relative amounts of demethylated *XIST* fragment; (**B**) relative amounts of the demethylated *XIST* fragment in high and low Johnsen’s score groups, respective ROC curve in (**C**); and (**D**) percentage of the demethylated *XIST* fragment in high and low Johnsen’s score groups, respective ROC curve in (**E**). **** indicates *p* < 0.0001. Abbreviations: AUC—area under the curve and JS—Johnsen’s score.

Given the knowledge on *XIST* expression during spermatogenesis around the time germ cells enter meiosis, we set out to study its promoter demethylation in two independent testicular parenchyma cohorts with diverse spermatogenesis efficiency as reported by the Johnsen’s score. The clinicopathological features of the testicular parenchyma discovery series are depicted in Supplementary Table S3. We found a significant and strong positive correlation between the relative levels of demethylated *XIST* promoter and the Johnsen’s score attributed by the pathologist (r_s_ = 0.75, *p* < 0.0001). Hence, we categorized our testicular parenchyma series into “Johnsen’s score <4” (premeiotic germ cells only) and “Johnsen’s score ≥4” (postmeiotic germ cells present). Samples with Johnsen’s score <4 showed almost absent demethylated *XIST* fragment (in all except one case), which was significantly lower as compared with those with a score ≥4 (*p* < 0.0001, Figure 8). Demethylated *XIST* fragment was able to discriminate among testicular parenchyma with a higher or lower (i.e., <4 or ≥4) Johnsen’s score with an AUC of 0.87 (Figure 8). This allowed for a good performance in discriminating between these two groups of spermatogenesis quality, namely a 100% specificity (Table 2).

We pursued validation of our findings in a second independent cohort (description of this cohort is depicted in Supplementary Table S4). The percentage of demethylated *XIST* was again significantly higher in testicular biopsies with a Johnsen’s score ≥4 as compared to those with scores <4 (*p* < 0.0001). It allowed for a good discrimination between these two subgroups, with an AUC of 0.89 (Figure 8), confirming our previous data.

## 3. Discussion

GCTs are remarkable for their intimate connection with developmental biology. They reflect stages of germ cell and embryonic development, and the (epigenetic) events that take place during these processes [1]. The more profound understanding of this link with biology propelled the recent changes in classification of these tumors [25], and also contributed to uncovering the disease biomarkers available to date [8]. We do believe that exploring this connection between development and GCTs is the way to go when it comes to finding novel clinically relevant biomarkers.

In this setting, the event of X chromosome inactivation by *XIST* is an attractive target, since besides its physiological occurrence in females (karyotypically XX) [26], it is also maintained in the case of TGCTs. This lncRNA (called X-inactive specific transcript) is mapped in a chromosomal region called X chromosome inactivation center (XIC) and, in females, its expression is activated when in *cis*, leading to a series of chromatin alterations that include methylation and histone modifications, resulting in silencing of the excessive X chromosome material (Supplementary Figure S3) [11,27]. Then, in females, the inactive *XIST* allele is found to be methylated, while the other copy is demethylated, allowing for *XIST* lncRNA expression and subsequent *XIST*-mediated silencing of the redundant X chromosome genes [28]. On the other hand, in males, *XIST* is not expressed (i.e., both alleles are found to be methylated) in somatic cells (given that there is no need for compensating for redundant chromosome dosage, being males karyotypically XY), while it is expressed in the germ cell lineage [17]. In addition, in TGCTs, *XIST* is found to be expressed in tumor cells given the supernumerary X chromosomes, due to the initial step of polyploidization. Looijenga and collaborators showed in 1997 [12] that all type II TGCTs exhibited *XIST* expression, and found it to be consistent with karyotypic alterations showing gain of chromosome X.

Subsequent works have demonstrated quite convincingly the existence of X chromosome gains in TGCTs, both SE and NS alike, and *XIST* expression in both tumor types [12,29]. Kawakami et al. reported a higher frequency of *XIST* expression in SE as compared to NS (85% vs. 25%) [29], although Looijenga et al. demonstrated *XIST* expression in all TGCTs, in a way consistent with the X chromosome content [12]. Following up on these works, and recognizing the potential to regulate *XIST* promoter by methylation, Kawakami and coworkers studied in depth the promoter of *XIST* by means of bisulfite sequencing, finding 56 CpG sites and dividing the 5′ end into regions I to VI [13]. As region IV was found to be frequently demethylated it was selected for further validation using conventional PCR, widely available at the time. Despite a limited cohort (n = 18 SE and n = 13 NS), they detected the demethylated *XIST* fragment in all SEs and in 92% of NSs. Moreover, they demonstrated detection of demethylated *XIST* in 71% and 55% of plasma samples from SE and NS patients, respectively. In a similar fashion, they also detected the methylated *XIST* fragment in several samples, always more frequently in NS as compared to SE (77% and 45% of NS tissues/plasma samples vs. 61% and 43% of SE tissues/plasma samples). Our results in two independent cohorts of 250 and 146 TGCT samples (the largest analyzed, thus far) are perfectly in line with these, and further extend them by the use of quantitative approaches, instead of mere “presence” or “absence” of methylated or demethylated signal. In our cohort, SEs disclosed a significantly lower relative amount of *XIST* methylated fragment (Figure 2) and a significantly higher relative amount or percentage of *XIST* demethylated fragment (Figure 4 and Figure 5), compatible with findings of Ushida et al. [14]. As well, this was maintained even when considering all individual histological subtypes, which was an analysis not performed thus far. Focusing on the relative methylated *XIST* fragment might be misleading since it might also be due to some contamination from somatic cells in the tissue such as lymphocytes. An issue that was demonstrated by us and others in previous works [23]. In spite of this limitation, SEs showed a significantly lower amount of methylated *XIST* fragment as compared to patients with no (T)GCTs, with NS or specifically with pure EC, allowing for a good discrimination power among these subgroups of patients (Figure 3, Table 1). The absence of associations with clinical variables might indicate that the methylation status of *XIST* relates more to the defined (developmental) biology of these tumors, limiting its value as a prognostic biomarker, although diagnostically of interest.

Previous works have shown expression of *XIST* in GCNIS, which was more easily detected with increasing proportion of GCNIS-affected parenchyma, seeming to reflect evolution in the extent of this precursor lesion [12]. Our results are in line with those findings, since demethylated *XIST* was detected in 16 of 17 tested samples. In addition, the percentage of demethylation was significantly higher in pure SEs as compared with GCNIS (Figure 6), which makes sense in that GCNIS is the direct precursor of SE, in line with the so-called default pathway [7].

Regarding (T)GCT cell lines, it has been demonstrated that they also show *XIST* expression (in consonance with multiple copies of the X chromosome), although with some variation among cell lines [12]. While Kawakami et al. found *XIST* expression in the SE-like cell line TCam-2 but not in the NS-like cell lines ITO-II and NEC8 [29], Looijenga et al. indeed observed *XIST* expression as well in the most commonly used and by far better characterized NS-derived cells (NCCIT, NT2, and 2102Ep) [12]. This is again in accordance with our data, showing that demethylated *XIST* was detected in all cell lines tested (both SE-like TCam-2 and NS-derived NCCIT, NT2, and 2102Ep), compatible with *XIST* expression, although with some variation in distinct cells (Figure 7). Our data show a significantly higher percentage of *XIST* demethylation in NT2 cells (around 70% to 80%) as compared to 2102Ep and NCCIT (around 40% to 50%). This is in line with the balance of X chromosome gains in these cells already described, with fluorescence in situ hybridization (FISH) analysis depicting a higher X chromosome content for NT2 (three chromosomes) as compared to the other cell lines (two chromosomes) [12]. The higher dosage of X chromosomes in the NT2 results is accompanied by a higher relative degree of *XIST* demethylation, allowing for inactivation of the extra X chromosome material.

As mentioned above, *XIST* expression in males is restricted to particular stages of the germ cell lineage, however, it is dependent on the extent of spermatogenesis. As demonstrated before, *XIST* is not expressed in the testis with only premeiotic cells or in the case of Sertoli-cell only syndrome, but starts to be expressed around the time germ cells enter meiosis [17,18]. Although some suggest this re-expression of *XIST* might be necessary for a healthy spermatogenesis, recent data have suggested a *XIST*-independent mechanism for X chromosome inactivation specifically during spermatogenesis [30,31]. Regardless of that, *XIST* expression was indeed not detected in atrophic testis parenchyma lacking spermatogenesis and GCNIS, but was detected in parenchyma with active spermatogenesis and epididymis [12]. To the best of our knowledge, the assessment of *XIST* methylation status has not been assessed in testis parenchyma samples for means of comparison with Johnsen’s score. Our results demonstrate frequent demethylation of *XIST* promoter in testis samples with active spermatogenesis, in the absence of GCNIS or overt TGCT. Indeed, no significant differences in the relative amounts of demethylated *XIST* fragment were witnessed between TGCTs and testicular parenchyma samples, in the discovery cohort (Supplementary Figure S1); however, this was seen at the expense of the samples with normal, active spermatogenesis, as indicated by a higher Johnsen’s score. When setting a cutoff at Johnsen’s score 4 (with scores of 1 to 3 corresponding to the absence of postmeiotic germ cells, i.e., complete atrophy/sclerosis, Sertoli-cell only, and spermatogonia only, respectively, and scores ≥4 already containing increasingly more postmeiotic germ cells, from spermatocytes to spermatids and spermatozoon), demethylated *XIST* fragment was almost absent in samples with scores <4 in our discovery cohort, consistent with no expression of *XIST*. In line with this, we set out to look for a correlation between the amount of demethylated *XIST* and the Johnsen’s score, which we found and proved to be a significant and strong positive correlation (Figure 8). Validation of our findings was again pursued; in both the discovery and validation independent cohorts the relative amount or percentage of demethylated *XIST* was significantly higher in cases regarded as having a Johnsen’s score ≥4 (i.e., where postmeiotic germ cells were identified by the pathologist on routine examination) (Figure 8). Importantly, the amount or percentage of demethylated *XIST* (assessed by two techniques, both regular quantitative methylation specific PCR (qMSP) using SYBR green dye and high-resolution melting (HRM) methylation-sensitive analysis) was able to discriminate patients with higher (≥4) and lower (<4) Johnsen’s score with good performance (AUC = 0.87–0.89, and remarkably 100% specificity in the discovery cohort).

## 4. Materials and Methods

### 4.1. Discovery Cohort

#### 4.1.1. Testicular Germ Cell Tumor and Testicular Parenchyma Samples Selection

A cohort (already described and validated in [25]) of TGCT patients consecutively diagnosed at the Portuguese Oncology Institute of Porto between 2005 and 2017 was included in the study. Hence, a total of 156 GCNIS-related (type II) TGCT patients were included. All patients were operated on and subsequently treated at this Institution by the same multidisciplinary team. Both clinical files and histological data were reviewed according to the most recent 8th edition of the American Joint Committee on Cancer (AJCC) staging manual [32] and the 2016 World Health Organization (WHO) Classification of Tumors of the Urinary System and Male Genital Organs, respectively [7]. Patients with metastatic disease were further categorized according to the IGCCCG prognostic system [33,34]. Follow-up was last updated on May 2019.

From these patients, formalin-fixed paraffin-embedded (FFPE) orchiectomy tissue samples (prior to any systemic treatment) were available, and representative blocks were selected by a TGCT-dedicated pathologist. A minimum of 80% tumor cellularity and absence of extensive necrosis were required in the selection of blocks. Consultation cases and cases without adequate histological material available were excluded. Importantly, individual tumor areas were carefully macro-dissected (in a strategy already reported in [35,36,37]) and each component of mixed tumors was considered as an independent sample for further analyses. Hence, a total of 250 independent tumor samples (106 SE, 56 EC, 36 YST, 11 CH, and 41 TE) were included in the study.

Additionally, a cohort of 54 patients undergoing orchiectomy for non-TGCT pathology (including inflammatory disease, benign neoplasms such as Leydig cell tumors or adenomatoid tumors, and surgical castration in the context of prostate cancer) were selected. Slides were reviewed by an uropathology-dedicated pathologist and a Johnsen’s score was attributed to each case, as described [20,21]. All slides were systematically screened for the presence of GCNIS (with immunohistochemistry aid if needed), which was absent in all cases. Representative FFPE blocks of the testicular parenchyma were selected.

Ten micrometer sections were then ordered for DNA extraction. This study was approved by the ethics committee of the Portuguese Oncology Institute of Porto (Comissão de Ética para a Saúde— CES-IPO-1-2018).

#### 4.1.2. DNA Extraction and Bisulfite Treatment

DNA was extracted from the FFPE samples using RNA/DNA Purification Plus Kit (Norgen, Thorold, ON, Canada), according to manufacturer’s instructions. The DNA quantification and purity were assessed in NanoDrop^TM^ Lite Spectophotometer. Sodium bisulfite treatment of 1000 ng of genomic DNA was performed using a EZ DNA Methylation GoldTM Kit (Zymo Research, Irvine, CA, USA), according to manufacturer’s protocol.

#### 4.1.3. Methylation Analyses

The qMSP was performed using the same primers for demethylated and methylated region IV of the XIST promoter reported by Kawakami et al. [13]. β-Actin (ACTB) was used for normalization as previously reported and using the same primers described in the literature [38]. DNA amplification was detected using SYBR Green-dye and a melting curve was generated to assess the presence of nonspecific PCR products and primer-dimer. Reactions were carried out in 96-well plates using an ABI 7500 Real Time PCR System (40 cycles). In brief, 1 µL of modified DNA, 5 µL of Xpert Fast SYBR (GRISP), ROX, a variable volume of primers (0.3 µL for methylated and demethylated XIST, 0.4µL for β-actin), and sterile bi-distilled water in a total volume of 10 µL were added to each well. Primer annealing temperature was optimized at 64 °C for methylated and demethylated XIST and 60 °C for β-actin. For quantification purposes, five serial dilutions (in duplicate) of previously bisulfite treated CpGenomeTM Universal Methylated DNA and Unmethylated DNA (Merck Millipore, Burlingtone, MA, USA) were included for methylated and demethylated XIST, respectively. A standard curve was then generated which allowed for relative quantification. Reaction efficiency was above 90% in all plates which allowed for interpolate comparisons. All experiments on FFPE samples were carried out in triplicate and two negative controls were included in each plate. Relative levels of methylated and demethylated XIST were determined using the formula: methylation level = (target gene/β-actin), which was then multiplied by 1000 for easier tabulation.

### 4.2. Validation Cohort

#### 4.2.1. Testicular Germ Cell Tumor and Testicular Parenchyma Samples Selection

A second, independent, validation cohort was included in the study. It included a cohort of frozen tissue from 146 TGCT patients (64 pure SE and 82 NS, comprising 38 mixed tumors, 30 pure EC, 4 pure YST, 4 pure CH, and 6 pure TE), and also 17 frozen tissue sections from GCNIS (without overt TGCT). Tissue samples were collected from several hospitals across the Netherlands and immediately frozen at −80 °C. All samples corresponded to orchiectomy specimens of patients not subjected to any systemic treatments. Sections were ordered for histological examination by the same experienced TGCT-dedicated pathologist (J. Wolter Oosterhuis).

Additionally, a total of 32 testis biopsies which had been performed for infertility issues were included. They corresponded to FFPE tissue which was reviewed by a uropathology-dedicated pathologist and a Johnsen’s score was attributed to each sample. No sample depicted GCNIS lesions.

Use of patient samples remaining after diagnosis was approved for research by the Medical Ethical Committee of the EMC (the Netherlands), permit no. 02.981. This included permission to use the secondary samples without further consent. Samples were used according to the ‘‘Code for Proper Secondary Use of Human Tissue in The Netherlands’’ developed by the Dutch Federation of Medical Scientific Societies (FMWV, version, 2002; update 2011).

#### 4.2.2. Cell Lines

The (T)GCT cell lines TCam-2, NT2, NCCIT, and 2102Ep were cultured as previously described in [39]. Briefly, they were cultured at 37 °C in a humidified cell-culture incubator with 5% CO_2_. TCam-2 was grown in RPMI and the remaining cell lines in DMEM media (both with 10% fetal bovine serum and 1% penicillin/streptomycin) (GIBCO, Invitrogen, Carlsbad, CA, USA).

#### 4.2.3. DNA Extraction and Bisulfite Treatment

The DNeasy Blood and Tissue Kit (Qiagen) and the phenol-chloroform method were used for DNA extraction from frozen tumors, cell lines, and FFPE samples. After that, sodium bisulfite treatment ranging from 500–1000 ng of genomic DNA was performed using EZ-96 DNA Methylation-Gold Kit (Zymo Research, Irvine, CA, USA), according to the manufacturer’s protocol.

#### 4.2.4. Methylation Analysis (High-Resolution Melting)

For the validation series, a more sensitive method was employed to improve preliminary results in the discovery cohort, and therefore the HRM methylation study [40,41] was performed to determine the precise percentage of demethylated *XIST* fragment. Bisulfite-specific primers for the region IV of the *XIST* promoter were designed using Methyl Primer Express^®^ Software (Applied Biosystems, Foster City, CA, USA), as indicated by the manufacturers (forward: 5′ TGTTGTTGATTATTTGGTGGT 3′ and reverse: 5′ TACCTCCCTAATTTAACTTAACACAA 3′). As in the previous experiment, β-actin was used as the control for bisulfite-treatment. The DNA amplification was detected using MeltDoctor™ HRM dye and a melting curve was generated. Real-time quantitative PCR (RT-qPCR) was performed in 96-well plates, using a QuantStudio 12K Flex Real-Time PCR System (ThermoFisher), with 40 cycles for fresh frozen tissue and cell lines and 50 cycles for FFPE. 1.25 µL (for fresh frozen tissue samples and cell lines) and 2.5 µL (for FFPE samples) bisulfite-treated DNA, 10 µL MeltDoctor™ HRM Master Mix, 0.6 µL of forward and reverse primers (10µM solutions), and sterile bi-distilled water in a total volume of 20 µL were added to each well. Primer annealing temperature was optimized at 63 °C. For each target sequence a no template control was included and male and female DNA samples (from peripheral blood lymphocytes) were included in each plate as additional controls. Importantly, the percentage of demethylated *XIST* was quantified using 0, 2%, 5%, 10%, 25%, 50% and 100% demethylated DNA standards that were generated by mixing different ratios of bisulfite-treated 100% methylated and 100% non-methylated Human HCT116 DKO DNA (ZymoResearch, Irvine, CA, USA), as recommended by the manufacturers.

### 4.3. Statistical Analysis

Data was tabulated using Microsoft Excel 2016 and analyzed using IBM SPSS Statistics version 24 and GraphPad Prism 6. Differences in relative methylation and demethylation among groups were assessed by the non-parametric Mann–Whitney U test and Kruskal–Wallis test, as appropriate. Cell lines were compared with unpaired t-test or ordinary one-way ANOVA, as appropriate. Associations between categorical variables were determined by means of a Chi-square test. All *p*-values were adjusted to multiple testing by Dunn’s or Turkey’s test, as appropriate. In patients diagnosed with mixed TGCT, the highest methylation value among all components was considered for assessing associations with clinicopathological features. Methylation data was correlated with patients’ age and Johnsen’s score using the Spearman non-parametric correlation test. Biomarker performance was assessed through receiver operating characteristics (ROC) curve construction. For both methylated and demethylated *XIST*, the Youden’s method [42,43] was used to achieve a cutoff to maximize the sensitivity and specificity. In addition, area under the curve (AUC), sensitivity, specificity, positive predictive value (PPV), negative predictive value (NPV), and accuracy were ascertained for both targets. Statistical significance was set at *p*-value < 0.05.

## 5. Conclusions

To conclude, we have provided evidence on the value of determining the *XIST* promoter methylation status both for diagnosis of TGCTs and for assessment of spermatogenesis extent. Some limitations of our work include its retrospective nature and the focus on tissue samples. However, we have gathered the largest cohort of TGCTs thus far focusing on studying *XIST* demethylation, and have replicated our data in a second, large independent cohort, reinforcing the strength of our results. In addition, we have employed two different techniques to achieve the same conclusions, including high-resolution melting methylation analyses, which improved the overall detection of *XIST* demethylated fragment.

Better TGCT biomarkers amenable for detection in liquid biopsies are needed to complement the current available serum tumor markers. On the basis of the results on the tissues discussed, we believe that detection of *XIST* demethylation is a promising candidate and we intend to pursue its detection in a large cohort of serum or plasma samples from TGCT patients. Moreover, Johnsen’s score is a laborious and limited methodology for assessing spermatogenesis in testicular biopsies and for clinical decision making. Given the correlation with spermatogenesis efficiency, assessment of *XIST* demethylation levels in semen samples from infertile patients will be of interest as a non-invasive way for better grouping of these patients and deciding on the best treatment option.

## Figures and Tables

**Table 1:** Discrimination performance parameters for *XIST* methylated fragment.

Context.	AUC (95% CI)	Sensitivity (%)	Specificity (%)	PPV (%)	NPV (%)	Accuracy (%)
SE vs. non-GCT cases	0.81 (0.74–0.88)	73.5	74.1	81.3	64.5	73.7
SE vs. NS cases	0.83 (0.76–0.89)	77.1	80.8	82.1	75.6	78.8
SE vs. EC cases	0.75 (0.54–0.95)	84.3	70.0	95.9	35.0	82.8

Abbreviations: AUC—area under the curve; CI—confidence interval; PPV—positive predictive value; NPV—negative predictive value.

**Table 2:** Performance parameters for *XIST* demethylated fragment in discriminating distinct Johnsen’s score categories.

Context.	AUC (95% CI)	Sensitivity (%)	Specificity (%)	PPV (%)	NPV (%)	Accuracy (%)
JS ≥ 4 vs. JS < 4	0.87 (0.77-0.97)	75.7	100	100	60.9	82.4

**Abbreviations:** AUC—area under the curve; CI—confidence interval; JS—Johnsen’s score; PPV—positive predictive value; NPV—negative predictive value.

## Supplementary Materials

The following are available online at https://www.mdpi.com/2072-6694/11/9/1385/s1: Supplementary Figure S1. Demethylated *XIST* fragment relative amounts in testicular germ cell tumors and testicular parenchyma of the discovery cohort. Relative amounts of the demethylated *XIST* fragment among testicular parenchyma samples and (A) all TGCT samples; (B) all SE samples; (C) only pure SE samples; and (D) all NS samples. Abbreviations: NS—non-seminoma; SE—seminoma; TGCT—testicular germ cell tumor; Supplementary Figure S2. Demethylated *XIST* fragment relative amounts in seminoma and embryonal carcinoma patients of the discovery cohort. ** indicates *p* < 0.01. Abbreviations: EC—embryonal carcinoma and SE—seminoma; Supplementary Figure S3. *XIST* methylation and expression and X chromosome inactivation in humans. (A) Methylation status of the *XIST* promoter in somatic male cells (XY, both allelles methylated, no expression), in female somatic cells (XX, there is *XIST* demethylation and expression), and in TGCTs (gains of X chromosome, there is *XIST* demethylation and expression) and (B) mechanism of X chromosome inactivation, starting with transcription of the *XIST* lncRNA (encoded in the X chromosome inactivation centers) which induces chromatin modifications resulting in silencing of redundant X chromosome genes. Abbreviations: XIC—X inactivation centers; Xa—activated X chromosome; Xi—inactivated X chromosome; Supplementary Table S1 Clinicopathological features of testicular germ cell tumor cases in the discovery cohort; Supplementary Table S2 Clinicopathological features of testicular germ cell tumor cases in the validation cohort; Supplementary Table S3 Clinicopathological features of non-germ cell tumor cases in the discovery cohort; Supplementary Table S4 Clinicopathological features of patients undergoing testicular biopsy for infertility issues in the validation cohort.
