# Supplementary material for: XIST-Promoter Demethylation as Tissue Biomarker for Testicular Germ Cell Tumors and Spermatogenesis Quality
Source: Cancers (Basel). 2019 Sep 17;11(9):1385. doi: 10.3390/cancers11091385 (PMC6769809; doi:10.3390/cancers11091385)
Supplement: Supplementary file 1 [file cancers-11-01385-s001.pdf]

cancers

Supplementary Materials: *XIST*-Promoter Demethylation as Tissue Biomarker for Testicular Germ Cell Tumors and Spermatogenesis Quality

Table 1. Clinicopathological features of testicular germ cell tumor cases in the discovery cohort.

Variables	Cohort (*n* = 156 cases)/Individual tumor samples (*n* = 250)
Age [years (median, IQR)]	30 (25–36)
Histologic subtypes (n, %)	
Seminoma	83 (53.2)
Embryonal carcinoma	10 (6.4)
Teratoma, postpubertal-type	3 (1.9)
Mixed tumor	60 (38.5)
Histologic subtypes detailed (n, %)	
Seminoma	106 (42.4)
Embryonal carcinoma	56 (22.4)
Yolk sac tumor, postpubertal-type	36 (14.4)
Choriocarcinoma	11 (4.4)
Teratoma, postpubertal-type	41 (16.4)
Stage	
I	101 (64.7)
II	31 (19.9)
III	24 (15.4)
IGCCCG group (for metastatic disease)	
Good	42 (76.4)
Intermediate	6 (10.9)
Poor	7 (12.7)

Abbreviations: IGCCCG—International Germ Cell Cancer Collaborative Group; IQR – interquartile range.

Table 2. Clinicopathological features of testicular germ cell tumor cases in the validation cohort.

Variables	Cohort (*n* = 146)
Age [years (median, IQR)]	30 (25–38)
Histologic subtypes (*n*, %)	
Seminoma	64 (43.9)
Mixed tumors	38 (26.0)
Embryonal carcinoma	30 (20.6)
Yolk sac tumor, postpubertal-type	4 (2.7)
Choriocarcinoma	4 (2.7)
Teratoma, postpubertal-type	6 (4.1)
Stage	
I	66 (69.5)
II	17 (17.9)
III	12 (12.6)
Variables	GCNIS cohort (*n* = 17)
Age [years (median, IQR)]	32 (24–35)

Abbreviations: IQR—interquartile

range.

Table 3. Clinicopathological features of non-germ cell tumor cases in the discovery cohort.

Variables	Cohort (*n* = 54)
Age [years (median, IQR)]	65 (42–77)
Johnsen score (*n*, %)	
0	1 (1.9)
2	3 (5.6)
3	12 (22.2)
4	2 (3.7)
5	1 (1.9)
6	1 (1.9)
7	2 (3.7)
8	8 (14.8)
9	15 (27.8)
10	9 (16.7)
Reason for orchiectomy (*n*, %)	
Surgical castration (prostate cancer)	11 (20.4)
Sex-cod/stromal tumor (Leydig, Sertoli)	5 (9.3)
Adenomatoid tumor	2 (3.7)
Inflammatory disease	9 (16.7)
Lymphoma/leukemia	5 (9.3)
Other	21 (38.9)

Abbreviations: IQR—interquartile range.

Table 4. features of patients undergoing testicular biopsy for infertility issues in the validation cohort.

Variables	Patient cohort (*n* = 32)
Age [years (median, IQR)]	33 (29–39)
Johnsen score (n, %)	
2–3	16
6–7	2
8–10	14

Abbreviations: IQR—interquartile range.

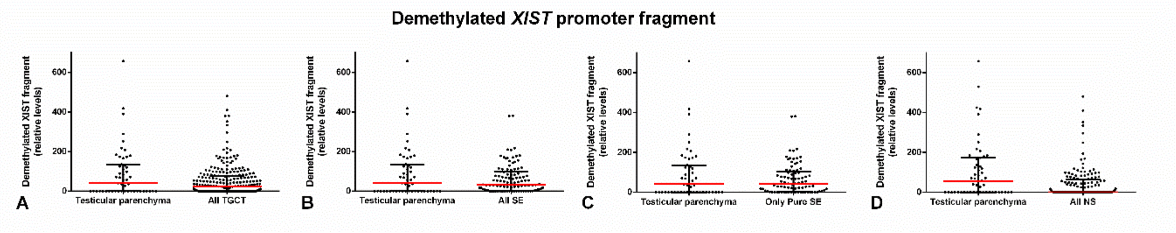

Supplementary Figure 1. Demethylated *XIST* fragment relative amounts in testicular germ cell tumors and testicular parenchyma of the discovery cohort. Relative amounts of the demethylated *XIST* fragment among testicular parenchyma samples and (A) all TGCT samples; (B) all SE samples; (C) only pure SE samples; and (D) all NS samples. Abbreviations: NS—non-seminoma; SE—seminoma; TGCT—testicular germ cell tumor.

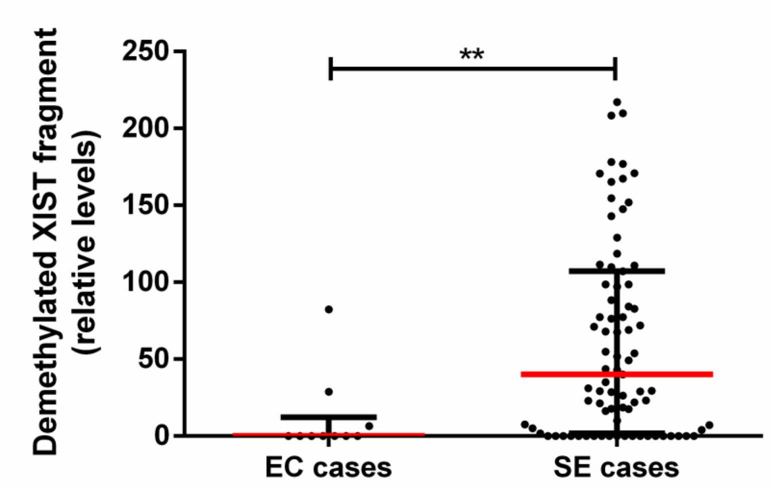

Supplementary Figure 2. Demethylated *XIST* fragment relative amounts in seminoma and embryonal carcinoma patients of the discovery cohort. ** indicates $p < 0.01$. Abbreviations: EC—embryonal carcinoma and SE—seminoma.

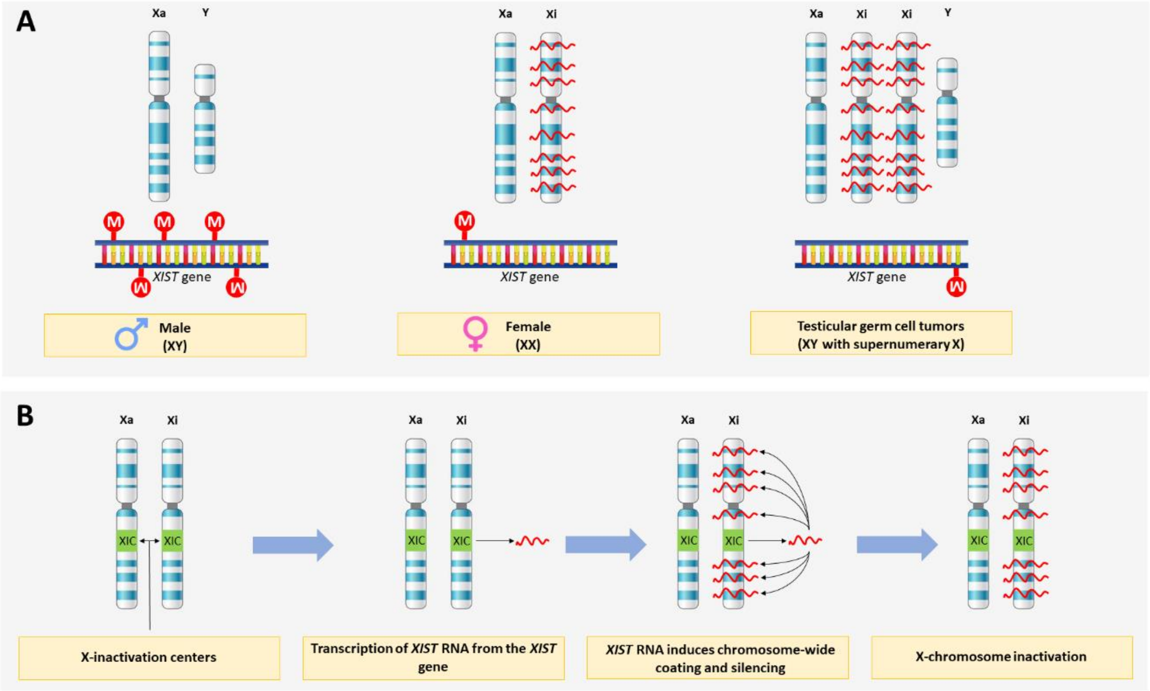

Supplementary Figure 3. *XIST* methylation and expression and X chromosome inactivation in humans. (A) Methylation status of the *XIST* promoter in somatic male cells (XY, both alleles methylated, no expression), in female somatic cells (XX, there is *XIST* demethylation and expression), and in TGCTs (gains of X chromosome, there is *XIST* demethylation and expression) and (B) mechanism of X chromosome inactivation, starting with transcription of the *XIST* lncRNA (encoded in the X chromosome inactivation centers) which induces chromatin modifications resulting in silencing of redundant X chromosome genes. Abbreviations: XIC – X inactivation centers; Xa – activated X chromosome; Xi – inactivated X chromosome.

© 2019 by the authors. Submitted for possible open access publication under the terms and conditions of the Creative Commons Attribution (CC BY) license (http://creativecommons.org/licenses/by/4.0/).
